# Complications of ovarian cancer surgery in Dr. Cipto Mangunkusumo National Referral Hospital, Jakarta: A cross-sectional study

**DOI:** 10.1016/j.amsu.2022.103581

**Published:** 2022-04-04

**Authors:** Gatot Purwoto, Boeyoeng Ego Dalimunthe, Aria Kekalih, Dita Aditianingsih, Yarman Mazni, Irfan Wahyudi, Kelli Julianti

**Affiliations:** a Department of Obstetrics and Gynecology, Faculty of Medicine University of Indonesia – Dr. Cipto Mangunkusumo National Referral Hospital, Jakarta, Indonesia; b Department of Community Medicine, Faculty of Medicine University of Indonesia, Jakarta, Indonesia; c Department of Anesthesiology and Intensive Care, Faculty of Medicine University of Indonesia – Dr. Cipto Mangunkusumo National Referral Hospital, Jakarta, Indonesia; d Department of Surgery, Faculty of Medicine University of Indonesia – Dr. Cipto Mangunkusumo National Referral Hospital, Jakarta, Indonesia; e Department of Urology, Faculty of Medicine University of Indonesia – Dr. Cipto Mangunkusumo National Referral Hospital, Jakarta, Indonesia

**Keywords:** Complications, Debulking, Ovarian cancer, Sepsis, Surgery

## Abstract

**Background:**

Ovarian cancer remains as one of the deadliest gynecologic problems globally. Often appears in advanced state, its surgery proves to be a challenge for clinicians. This study aim to present complications surrounding ovarian cancer surgery.

**Materials and methods:**

This study was a cross-sectional study to analyze reports of intraoperative and postoperative complications in ovarian cancer patients undergoing laparotomy in Dr. Cipto Mangunkusumo National General Hospital, Jakarta from January 2018 to December 2019. Ovarian cancer patients undergoing laparotomy surgery were included in the study. Patients with a history of other cancers or having incomplete data were excluded from the study. Intraoperative complications included intestinal, ureter, bladder injury, and postoperative complications included paralytic ileus, surgical wound infection and sepsis were documented.

**Results:**

A total of 78 subjects were included in the study. The total proportion of complications was 19.2%. The most prevalent intraoperative complications were intestinal injury (12.8%), bladder injury (2.6%), and ureter injury (1.3%). Most prevalent postoperative complications reported were surgical wound infection (5.2%), sepsis (3.9%), while none of the patients had paralytic ileus.

**Conclusion:**

The proportion of intraoperative and postoperative complications in ovarian cancer surgery was still at alarming level (19.2%). Further steps are needed to ameliorate the rate of complications surrounding ovarian cancer surgery.

## Highlights

• Ovarian cancer remains as one of the deadliest gynecologic problems globally.
• The proportion of intraoperative and postoperative complications in ovarian cancer.
• Reducing the incidence of surgical complications would improve outcome quality and reduce the cost of hospitalization.

## 1 Introduction

Ovarian cancer is the second most common gynecological malignancy in the world^.^ [1] Previous data in the UK indicated that there are approximately 6500 new cases of ovarian cancer per year. Most of these cases are found at an advanced stage resulting in poor overall prognosis [1]. The 5-year survival rate for early-stage ovarian cancer is around 90%, while the 5-year survival rate for advanced ovarian cancer is 10%–30% [2].

Women with suspicion of ovarian cancer would generally undergo surgery, either to confirm the diagnosis, find the extent of disease spread (surgical staging), or incomplete/complete tumor removal [3]. The basic surgical procedures used in the management of advanced ovarian tumor include primary cytoreduction, secondary cytoreduction, exploration by biopsy, interval cytoreduction, laparotomy, or laparoscopy. The role of cytoreduction in ovarian cancer has been investigated for more than 50 years and is currently considered the standard management of primary ovarian cancer surgery, which is usually followed by chemotherapy [4].

Surgical complications have been shown to be related to admission time which ultimately translates into cost [5]. Surgical complications vary widely depending on the anatomy and location of the tumor. Abdominal surgery is associated with a high complication rate due to the innate anatomical structure of the ovaries located in the abdominal cavity [6]. Therefore, reducing the incidence of complications due to surgery is very important in improving the quality of outcome and simultaneously reducing the cost of hospitalization [7].

Unfortunately, data regarding postoperative complications in ovarian cancer patients are not widely available, especially in Indonesia. In fact, these data are very important as an initial step in determining the direction of prevention and management of surgical complications in ovarian cancer patients. It is expected that this study would further signify complications regarding ovarian cancer surgery and help to determine the steps needed to be initiated in order to ameliorate the outcome for patients.

## 2 Methods

This study was a cross-sectional study to analyze reports of intraoperative and postoperative complications in ovarian cancer patients undergoing laparotomy at Dr. Cipto Mangunkusumo National Referral Hospital, Jakarta from January 2018 to December 2019. This study use 5% error bound and 95% confidence interval limit, power of the test considered to be 90%. This paper was registered in a research registry with the unique identifying number (UIN) of 7679 (https://www.researchregistry.com/browse-the registry#home/registrationdetails/6217a671cbb7bb002073f02e/) and has been reported in line with the STROCSS criteria [14].

**Fig. 1:**
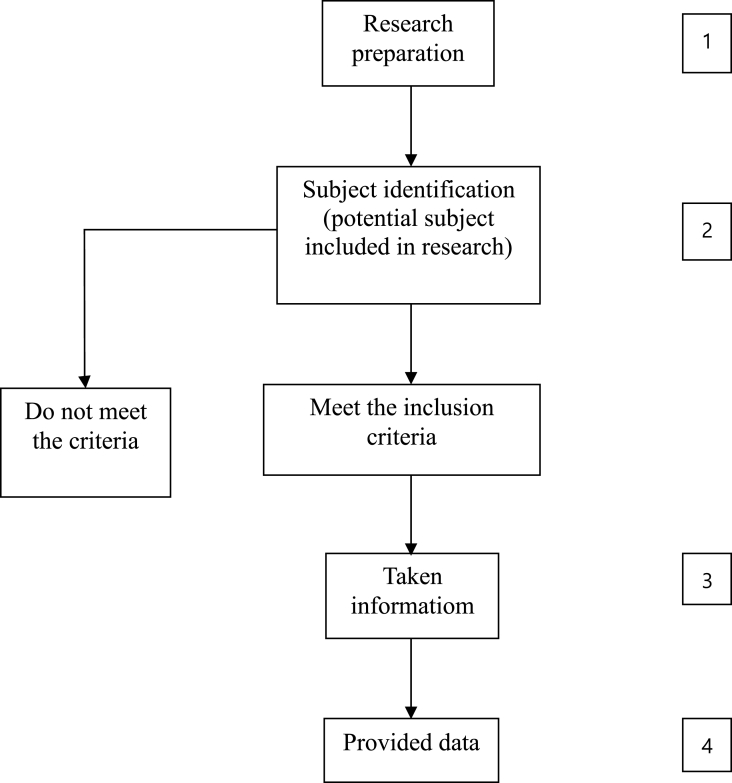
Flow of participants.

Ovarian cancer patients undergoing laparotomy surgery for either surgical staging, cytoreduction, or excision were included in the study. Patients with history of other primary carcinoma or having incomplete data were excluded from the study. Data regarding characteristics and treatments received were taken from medical record (Fig. 1).

The intraoperative complications included in this study were intestinal injury, ureter injury, and bladder injury. Postoperative complications included in this study were sepsis, paralytic ileus, and surgical wound infection. Those complications were determined by surgery operator intraoperatively or inpatient consultants assigned.

Characteristics analyzed in this study were age, body weight, The International Federation of Gynecology and Obstetrics (FIGO) stage, preoperative hemoglobin level, intraoperative adhesion, operator's experience, surgery duration, and intraoperative bleeding amount.

The study was approved by the Faculty of Medicine, University of Indonesia. All human studies have been approved by the Research Ethics Committee on ethical approval letter numbered KET-148/UN2.F1/ETIK/PPM.00.02/2019. Due to the retrospective nature of this study, informed consent was not signed by each participant. However, there was no identifying variables included in this study.

Collected data are analyzed using SPSS for Macintosh ver. 20. Characteristics of patients were analyzed descriptively. Bivariate and multivariate analysis was used to assess risk factors of any surgical complication (either intraoperative or postoperative).

## 3 Results

A total of 97 patients met the inclusion criteria. However, 19 of whom had incomplete medical record and was excluded. Therefore, 78 subjects were further analyzed.

Univariate test was performed to assess the general characteristics of the study subjects’ sociodemographic and clinicopathologic variables (Table 1).

**Table 1:** Sociodemographic and clinicopathologic characteristics of patients.

Characteristics	n = 78
Stage (FIGO)	
1	30 (38.5%)
2	14 (17.9%)
3	23 (29.5%)
4	11 (14.1%)
Intraoperative adhesion	
Yes	63 (80.8%)
No	15 (19.2%)
Operator's experience	
< 10 years	49 (62.8%)
>10 years	29 (37.2%)
Operation duration^a^	300 (120–567)
< 5.5 h	49 (62.8%)
> 5.5 h	29 (37.2%)

* Mean ± deviation standard.

a Median (range).

Following the characteristics of subjects, intraoperative and postoperative complications was counted and analyzed. The results can be found on Table 2.

**Table 2:** Complications during ovarian surgery.

Complication	n	Percentage	CI 95%
Intraoperative			
Intestinal injury	10/78	(12.8%)	5.4–20.2%
Ureter injury	1/78	(1.3%)	0.3–3.8%
Bladder injury	2/78	(2.6%)	0–6.1%
Postoperative			
Sepsis	3/78	3 (3.9%)	0–8.1%
Surgical site infection	4/78	4 (5.2%)	0.2–10%

Based on the analysis, the most common surgical complication for subjects was intestinal surgery, followed by surgical site infection and sepsis.

Furthermore, subjects were included in the complication (+) group if they had a minimum of 1 complication (either intraoperative or postoperative). The result of bivariate analysis can be found on Table 3.

**Table 3:** Bivariate analysis of surgical complications risk factors.

Characteristics	Complication (a)	Complication (−)	p
FIGO stage			
1	4 (13.3%)	26 (86.7%)	Control
2-4	11 (22.9%)	37 (77.1%)	0.242
Intraoperative adhesion			
Yes	13 (20.6%)	50 (79.4%)	Control
No	2 (13.3%)	13 (86.7%)	0.519
Operator's experience			
< 10 years	5 (10.2%)	44 (89.8%)	Control
> 10 years	10 (34.4%)	19 (65.6%)	**0.008**
Surgery duration^a^	360 (120–567)	290 (120–510)	
< 5.5 h	46 (93.9%)	3 (6.1%)	Control
> 5.5 h	17 (58.6%)	12 (41.4%)	**< 0.001**

* Mean ± deviation standard.

a Median (range).

Following the bivariate analysis, multivariate analysis was done in order to determine surgical complications’ risk factors. The result can be found on Table 4.

**Table 4:** Multivariate analysis of surgical complications risk factors.

Variables	OR	95%CI	P
Operator's experience >10 years	2.183	1.172–4.065	0.014
Surgery duration >5.5 h	4.271	1.043–17.49	0.044
Constant	0.001	–	0.002

## 4 Discussion

In this study, the proportion of complications was divided into intraoperative complications and postoperative complications. Intraoperative complications were divided into 3 categories, namely intestinal injury, ureter injury, and bladder injury. Bowel injury was the complication with the highest proportion in this study with proportion reaching 12.8%. The jejunum and ileum are the most common sites for intestinal injury in gynecological surgery. Although colon injuries are less common, postoperative complications resulting from colon injuries are more common. Based on previous research, it was one of the most common iatrogenic injuries in gynecological cancer surgery [8]. Bowel injuries that occur during surgery can progress to include perforation, peritonitis, abscess formation, intestinal obstruction, and fistula formation.

Furthermore, ureter injury occurred in 1 (1.3%) of all subjects, whereas bladder injury occurred in 2 (2.6%) of all subjects. These results were similar to previous studies which stated that the prevalence of urinary tract injury was 3%, with the prevalence of bladder injury up to five times the prevalence of ureteral injury.^8^ Urinary tract injury is a type of iatrogenic injury that often occurs in obstetrics and gynecology. Injury to the ureter often occurs due to misidentification of the ureter as a blood vessel, especially in operations performed in an emergency. Another condition that can occur is the ureter that crosses the tumor mass so that it needs to be resected, resulting in imperative iatrogenic injury. Injuries to the urinary tract often go unnoticed at the time of surgery, with a mean of 26%–95% of injuries identified postoperatively [8]. In the case of bladder injuries, intraoperative identification and immediate repair are essential to prevent leakage of urine that can be resulting in peritonitis to sepsis [8].

It was found that 3 (3.9%) subjects experienced sepsis during the inpatient monitoring period. This figure is higher than a study conducted in America of 1.2% [9]. In that study, it was said that the prevalence of sepsis would increase with increasing patient age as well as in certain races. However, previous study used more samples with Caucasians who had a lower prevalence of sepsis than other races [9]. Sepsis is one of the highest causes of postoperative death with a mortality rate of up to 20.8% [10].

A total of 4 (5.2%) subjects experienced surgical wound infection during the follow-up period. Although the prevalence of this condition appears to be quite high, this value is lower than in similar studies with a prevalence of 10–15% of all ovarian cancer operations [11]. Based on previous studies, surgical wound infection in ovarian cancer surgery is mostly deep incisional and deep types [11]. There are several factors that are known to be associated with an increase in surgical wound infection in ovarian cancer cases, such as extensivity of the procedure, complexity of action, comorbid factors such as smoking and peripheral artery disease, and intraoperative bleeding.

There was a result obtained in this study which was quite peculiar. In a previous study by Bilimoria et al., it was found that operators with higher experience or having a higher level of specialization would increase the subject's outcome [12]. However, in this study it was known that the opposite occurred, namely a higher complication rate was found in operators with higher levels of experience. This could occur due to various factors, one of which was the location of the research. In previous studies that analyzed the relationship between operator's experience and patient outcomes, data collection was carried out in multiple centers [12]. However, this study was taken in teaching hospitals with diverse competencies. Therefore, patients with a low likelihood of complications will be managed by an operator with less experience, with close supervision by a consultant gynecologist oncology while more difficult surgery would be handled directly by consultants. This would ultimately gave a tendency of higher complication rate for operators with more experience. One study by Kumar et al. added a variable of “operation complexity” to describe this problem [13]. Unfortunately, this study had not identified this variable so that it cannot be carried out in further analysis.

We could concluded that the proportion of intraoperative and postoperative complications in ovarian cancer surgery was still at alarming level (19.2%). Further steps are needed to ameliorate the rate of complications surrounding ovarian cancer surgery.

## 5 Conclusion

The proportion of intraoperative and postoperative complications in ovarian cancer surgery was still at alarming level (19.2%). Further steps are needed to ameliorate the rate of complications surrounding ovarian cancer surgery.

## Availability of data and materials

The datasets used and/or analyzed during the current study are available from the corresponding author on reasonable request.

## Declaration of competing interest

There is no conflict of interest among the participants of the article.

## Ethical approval

This study has been given ethical clearance from Faculty of Medicine University of Indonesia ethical committee number KET-148/UN2.F1/ETIK/PPM.00.02/2019.

## Sources of funding

This work did not receive any grant from funding agencies in the public, commercial or non-for-profit sectors.

## Author contribution

GP performed conceptualization; BED conducted acquisition and analysis of data; AK conducted statistics and analysis of data; DA conducted discussion and conception in Anesthesiology Field; YM conducted discussion and conception in Surgery Field; IW conducted discussion and conception in Urology Field; KJ managed the draft of manuscript and coordination.

## Trial registry number

The name of the registry: Complications of Ovarian Cancer Surgery in Dr. Cipto Mangunkusumo National Referral Hospital, Jakarta.

Unique Identifying Number (UIN): researchregistry7679.

Hyperlink:https://www.researchregistry.com/browse-the-registry#home/registrationdetails/6217a671cbb7bb002073f02e/

## Guarantor

Gatot Purwoto, MD, PhD, Oncologic-Gynecology Consultant.

Department of Obstetrics and Gynecology, Faculty of Medicine University of Indonesia - Dr Cipto Mangunkusumo National Referral Hospital, Jakarta, Indonesia.

Jl. Diponegoro 71, Jakarta. 10310.

E-mail: gatotpurwoto@gmail.com.

Phone: +62 816 1364 182.

## Consent

This study has been given ethical clearance from Faculty of Medicine University of Indonesia ethical committee number KET-148/UN2.F1/ETIK/PPM.00.02/2019. This study also promotes and ensures respect for all identified individuals and protect their health and rights. For research, this study used anonymized information about patients that they cannot be identified. Authors would like to express sincere gratitude to all participating patients who willingly support this study.

## Provenance and peer review

Not commissioned, externally peer reviewed.

## Declaration of competing interest

Author GP, Author BED, Author AK, Author DA, Author YM, Author IW, Author KJ declare that they have no conflict of interest.
